# Altered Brain Activity and Functional Connectivity in Unilateral Sudden Sensorineural Hearing Loss

**DOI:** 10.1155/2020/9460364

**Published:** 2020-09-22

**Authors:** Jiawei Chen, Bo Hu, Peng Qin, Wei Gao, Chengcheng Liu, Dingjing Zi, Xuerui Ding, Ying Yu, Guangbin Cui, Lianjun Lu

**Affiliations:** ^1^Department of Otolaryngology Head and Neck Surgery, Tangdu Hospital, Fourth Military Medical University, Xi'an 710038, China; ^2^Department of Radiology & Functional and Molecular Imaging Key Lab of Shaanxi Province, Tangdu Hospital, Fourth Military Medical University, Xi'an 710038, China; ^3^Student Brigade, Fourth Military Medical University, Xi'an 710032, China

## Abstract

**Background:**

Sudden sensorineural hearing loss (SSNHL) is an otologic emergency and could lead to social difficulties and mental disorders in some patients. Although many studies have analyzed altered brain function in populations with hearing loss, little information is available about patients with idiopathic SSNHL. This study is aimed at investigating brain functional changes in SSNHL via functional magnetic resonance imaging (fMRI).

**Methods:**

Thirty-six patients with SSNHL and thirty well-matched normal hearing individuals underwent resting-state fMRI. Amplitude of low-frequency fluctuation (ALFF), fractional ALFF (fALFF), and functional connectivity (FC) values were calculated.

**Results:**

In the SSNHL patients, ALFF and fALFF were significantly increased in the bilateral putamen but decreased in the right calcarine cortex, right middle temporal gyrus (MTG), and right precentral gyrus. Widespread increases in FC were observed between brain regions, mainly including the bilateral auditory cortex, bilateral visual cortex, left striatum, left angular gyrus (AG), bilateral precuneus, and bilateral limbic lobes in patients with SSNHL. No decreased FC was observed.

**Conclusion:**

SSNHL causes functional alterations in brain regions, mainly in the striatum, auditory cortex, visual cortex, MTG, AG, precuneus, and limbic lobes within the acute period of hearing loss.

## 1. Introduction

Hearing loss is one of the most common sensory disorders in the world. According to the World Health Organization, there are approximately 470 million patients with disabling hearing loss worldwide [1]. Sudden sensorineural hearing loss (SSNHL) is an otologic emergency that is defined as a sensorineural hearing decline of ≥30 dB in at least three consecutive frequencies in the pure-tone audiogram within 72 hours [2]. SSNHL is usually unilateral, with bilateral involvement accounting for less than 5% of the cases [3]. Recent population-based studies reported that the incidence of SSNHL ranges from 5 to 27 per 100,000 with a rapidly increasing annual incidence [4, 5]. The inner ear hair cells are mainly responsible for transducing sound vibrations into electrical impulses, and then these electrical signals are transmitted by spiral ganglion neurons (SGNs) into the brainstem to have the hearing function [6–10]. Thus, most cases of hearing loss are due to the damage or malfunction of hair cells or SGNs, both of which are sensitive and vulnerable to noise, aminoglycosides, chemotherapy regimens, inflammation, biological aging, and genetic defects [11–17]. Although some pathophysiological hypotheses have been proposed, the exact mechanisms of SSNHL remain unclear. Moreover, most of the studies on SSNHL focused on the hair cells and SGNs, while the alterations in the intensity and synchronization of brain activity after SSNHL have rarely been investigated. Auditory deprivation may impose a substantial socioeconomic burden on patients. Those who do not recover normal hearing have difficulties in sound localization and social communication, which may cause potentially mental disorders such as anxiety and depression [18, 19]. Therefore, studies that are aimed at elucidating the changes and underlying mechanisms of higher-order brain functions in deaf individuals are necessary.

Previous studies have reported alterations in the activity and functional organization of the cerebral cortex in subjects with unilateral hearing loss (UHL). Patients with long-term unilateral hearing impairment exhibited altered connectivity with other sensory and higher-order networks within and beyond the auditory network, reflecting a cross-modal functional reorganization in UHL [20]. Plasticity of the cortical tonotopic map within the primary auditory cortex has also been observed in patients with unilateral sensorineural hearing loss and tinnitus [21]. Another connectome analysis showed altered connections and nodal centrality in several brain functional networks in patients with UHL caused by acoustic neuromas [22]. These studies showed evidence of cortical functional reorganization following UHL. However, there have been limited studies [23, 24] available on the differences in brain function between unilateral SSNHL patients and normal hearing people. The changes in cortical activity and functional organization following SSNHL remain largely unknown.

Functional magnetic resonance imaging (fMRI) is a noninvasive technique for investigating the changes in brain function in many disorders, which could provide valuable information for elucidating the pathogenesis and guiding clinical practice. Based on the blood-oxygenation-level-dependent (BOLD) signals, fMRI has been reported to reflect underlying neuronal activity [25]. Analyses of the amplitude of low-frequency fluctuations (ALFF) and functional connectivity (FC) are two important methods used in fMRI studies. ALFF is defined as the amplitude of brain BOLD signal fluctuations in the low-frequency range (0.01-0.08 Hz), which could reflect the spontaneous neuronal activity of the cerebral cortex [26]. The analysis of FC calculates the temporal correlation of BOLD signal fluctuations between spatially remote brain regions. Positive FC indicates activity synchronization and functional correlation between two voxels or brain areas [27]. These two methods have been widely applied to explore alterations in the activity and organization of the brain in hearing loss and other diseases, including depressive disorder, Alzheimer's disease, and schizophrenia [28–32]. Analyses of ALFF and FC simultaneously allow researchers to explore the synchronization between brain regions and the activation of each brain region. Although a large number of fMRI studies have been performed in individuals with UHL, the changes in cortical function during the acute period of SSNHL have rarely been investigated.

To address these questions, we conducted ALFF, fractional ALFF (fALFF), and FC analyses with resting-state fMRI in patients with SSNHL and normal hearing people. We hypothesized that SSNHL would change the intensity and synchronization of cerebral activity.

## 2. Materials and Methods

### 2.1. Participants

Thirty-six patients with mild to profound unilateral SSNHL were recruited from the Department of Otolaryngology Head and Neck Surgery of Tangdu Hospital. Patients with UHL ≥ 30 dB in at least three consecutive frequencies within 72 hours were diagnosed with unilateral SSNHL according to the 2019 Sudden Hearing Loss Clinical Practice Guideline of the American Academy of Otolaryngology-Head and Neck Surgery Foundation (AAO-HNSF) [2]. Pure-tone audiometry was performed to calculate the pure-tone average (PTA) and assess hearing thresholds. Thirty sex- and age-matched normal hearing participants were recruited from the local community. The exclusion criteria included conductive hearing loss, Ménière's disease, acute or chronic otitis media, and central nervous system disorders such as brain trauma, tumors, and cerebrovascular disease. The data from scans of six SSNHL participants were excluded for excessive head movement. Finally, 30 unilateral SSNHL and 30 healthy control (HC) participants were recruited in our study. All the SSNHL and HC participants were right-handed. All the participants provided written informed consent, and the study protocol was approved by the ethics committee of Tangdu Hospital of the Air Force Medical University. Intergroup comparisons of age and body mass index (BMI) were conducted using two-tailed, two-sample Student's *t*-tests with SPSS 23 software (IBM, Armonk, NY, USA). In SSNHL patients, the differences in PTA before and after treatment were compared using two-tailed, paired-sample Student's *t*-tests. The significance level was set at *p* < 0.05.

### 2.2. MRI Data Acquisition

The MRI data were acquired before patients received any treatment. MRI scans were performed at the Radiology Department of Tangdu Hospital using a GE Discovery MR750 3.0 T scanner (General Electric Healthcare Systems, Boston, MA, USA) with an eight-channel phased-array head coil. Foam paddings were used to restrict head motion, and ear plugs were used to reduce scanner noise. During the data acquisition period, the participants were told to stay awake with their eyes closed in the scanner. Structural images including high-resolution T1-weighted images were acquired by using a three-dimensional brain volume (3D-BRAVO) sequence with the following parameters: echo time (TE) = 3.2 ms, inversion time (TI) = 450 ms, repetition time (TR) = 8.2 ms, flip angle (FA) = 12°, field of view (FOV) = 256 × 256 mm^2^, acquisition matrix = 256 × 256, slice thickness = 1.0 mm, and slice number = 188.

BOLD images were acquired by using a gradient-recalled echo-echo-planar imaging (GRE-EPI) sequence with the following parameters: TE = 30 ms, TR = 2000 ms, time points = 185, FA = 90°, FOV = 220 × 220 mm^2^, acquisition matrix = 64 × 64, slice thickness = 3 mm, slice number = 36, interslice gaps = 4 mm, and in − plane spatial resolution  = 3.4375 × 3.4375 mm^2^.

### 2.3. MRI Data Preprocessing

Functional MRI data were preprocessed using Data Processing and Analysis for Brain Imaging (DPABI) [33] and Statistical Parametric Mapping (SPM12) software (https://www.fil.ion.ucl.ac.uk/spm/software/spm12/) in the MATLAB R2014a platform (MATLAB 2014a, Mathworks, Inc, Natick, MA). The first 10 time points were discarded. The remaining images underwent a preprocessing procedure including slice timing and head motion correction, normalization to the Montreal Neurological Institute (MNI) space, linear trend removal, nuisance covariate regression, bandpass filtering (0.01-0.08 Hz), and smoothing with a 6 mm full width at half maximum (FWHM) isotropic Gaussian kernel. Any image with head motion > 3 mm translation or 3° rotation in any direction was excluded.

### 2.4. ALFF and fALFF Analysis

ALFF and fALFF were analyzed using the Data Processing Assistant for Resting-State fMRI (DPARSF) software [34]. The time series were converted into the frequency domain to obtain the power spectrum. ALFF was obtained by calculating the mean square root of the power spectrum of the signal with a frequency window of 0.01-0.08 Hz. To obtain the fALFF, the ratio of the power spectrum across 0.01-0.08 Hz to that across the entire frequency range was calculated. The ALFF and fALFF values were compared between the SSNHL and HC groups using two-tailed, two-sample Student's *t*-test with SPM12 software after regressing out nuisance covariates including sex, age, and BMI. Gaussian random field (GRF) correction for multiple comparisons was used, because it corrects the false positive rate at both the voxel and cluster levels. The significance levels were set at voxel < 0.005 and cluster < 0.025, as one recent study showed that this level effectively reduced the false positive rate below 0.05 [35].

### 2.5. Voxel-Wise FC Analysis

FC analysis was performed using SPM12 and RESting-state fMRI data analysis Toolkit (REST 1.6) [36]. Forty-four of the 112 brain regions in the Harvard-Oxford Atlas were selected as regions of interest (ROIs). The mean time series signal of each brain region was calculated. Correlation coefficients were calculated between the mean time series signal of each ROI and that of voxels across the whole brain. The correlation coefficients of each voxel were *Z*-scored to improve normality. Then, the resulting values of all voxels were compared between the SSNHL and HC groups to identify the brain areas with significant differences in FC. GRF was used for multiple comparison corrections. The results were visualized based on the Ch2 brain template using DPABI software.

### 2.6. ROI-Wise FC Analysis

Correlation coefficients between 44 selected ROIs and all 112 brain regions in the Harvard-Oxford Atlas for each participant were transformed with the Fisher's *r* to *z* method. Using SPSS 23 software, intergroup comparisons were performed with one-way ANOVA and the post hoc Dunnett's *t*-test. Multiple comparisons were corrected with both false discovery rate (FDR) and network-based statistic (NBS) methods [37]. The significance level was set at *p* < 0.05. FC with intergroup significant differences was visualized using BrainNet Viewer software [38].

## 3. Results

### 3.1. Demographic and Clinical Information

The demographic and clinical information of both groups are presented in Table 1. There were no significant differences in age or BMI between the SSNHL and HC groups. In patients with SSNHL, an average of 15 dB HL improvement was observed in the PTA of the affected ear after treatment. The difference in the PTA of the affected ear before and after treatment was significant (*p* < 0.001). The significant difference (*p* = 0.043) in the PTA of the unaffected ear before and after treatment may come from the measurement deviation of the pure-tone audiometry test.

### 3.2. Voxel-Wise ALFF and fALFF Analysis

**Figure 1:**
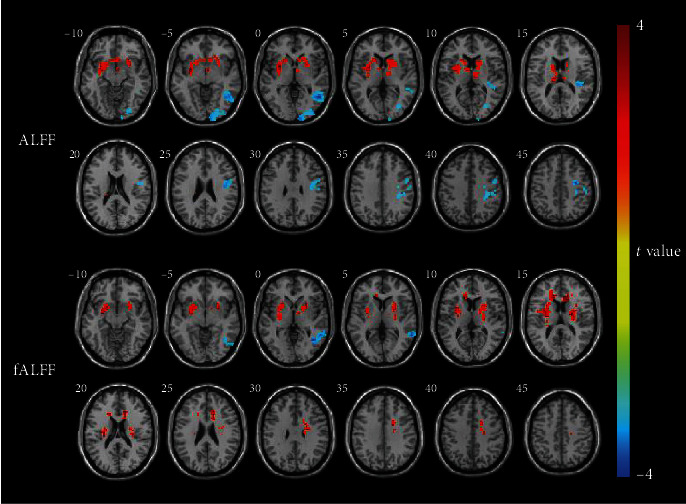
Intergroup comparison of ALFF and fALFF between the SSNHL and HC groups. The ALFF value of the SSNHL group was significantly increased in a cluster located in the left putamen but was decreased in three clusters located in the right calcarine cortex, right MTG, and right precentral gyrus compared with the HC group. The fALFF value in the SSNHL group was significantly increased in two clusters located in the right and left putamen but was decreased in a cluster located in the right MTG. Significantly increased ALFF or fALFF values are indicated in red, while significantly decreased ALFF or fALFF values are indicated in blue.

Intergroup comparisons showed differences in ALFF and fALFF between the SSNHL and HC groups (Figure 1). The ALFF value in the SSNHL group, compared with that in the HC group, was significantly increased in a cluster located in the left putamen. The ALFF value was significantly decreased in three clusters located in the right calcarine cortex, right middle temporal gyrus (MTG), and right precentral gyrus. fALFF values were significantly increased in two clusters located in the right and left putamen. The fALFF value was significantly decreased in a cluster located in the right MTG. The peak MNI coordinates and peak intensities of these clusters are presented in Table 2.

### 3.3. Voxel-Wise FC Analysis

**Figure 2:**
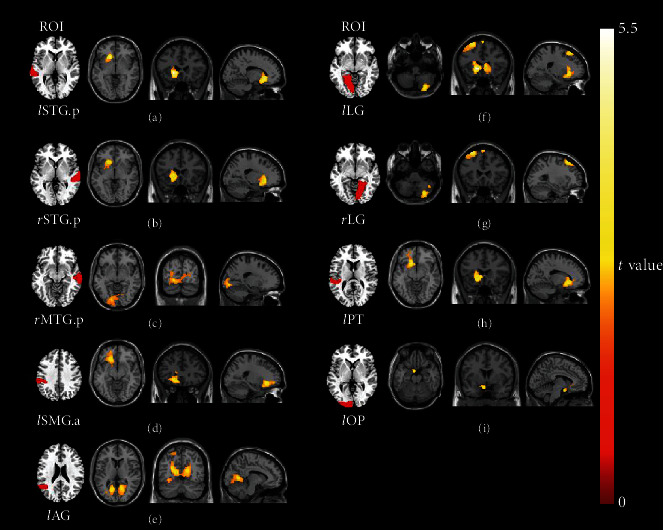
Intergroup comparisons of voxel-wise FC between the SSNHL and HC groups. Compared with the HC group, the SSNHL group showed significantly increased voxel-wise FC values in nine ROIs, including (a) the *l*STG.p and a cluster located in the left putamen and left caudate nucleus, (b) the *r*STG.p and a cluster located in the left putamen and left caudate nucleus, (c) the *r*MTG.p and a cluster located in the bilateral lingual gyri and the right calcarine cortex, (d) the *l*SMG.a and a cluster located in the left inferior frontal gyrus, (e) the *l*AG and a cluster located in the bilateral calcarine cortex and left posterior cingulate cortex, (f) the *l*LG and three clusters located in the right cerebellum posterior lobe, left caudate nucleus, and left MFG, (g) the *r*LG and two clusters located in the right cerebellum posterior lobe and left MFG, (h) the *l*PT and a cluster located in the left caudate nucleus and left putamen, and (i) the *l*OP and a cluster located in the left caudate nucleus and left putamen. The color bar indicates the *t*-value of voxels with significant intergroup differences. STG: superior temporal gyrus; MTG: middle temporal gyrus; SMG: supramarginal gyrus; AG: angular gyrus; LG: lingual gyrus; PT: planum temporale; OP: occipital pole; *l*: left; *r*: right; a: anterior division; p: posterior division.

Among the intergroup comparisons, we found 9 ROIs with significantly increased FC with other brain clusters in the SSNHL group (Figure 2). No decreased FC was observed. Compared with the HC group, the SSNHL group showed increased FC mainly between the bilateral auditory cortices and left striatum, the left visual cortex and left striatum, the right MTG and bilateral visual cortices, and the bilateral lingual gyri (LG) and left middle frontal gyrus (MFG). The peak intensity and MNI coordinates of these clusters are presented in Table 3.

### 3.4. ROI-Wise FC Analysis

**Figure 3:**
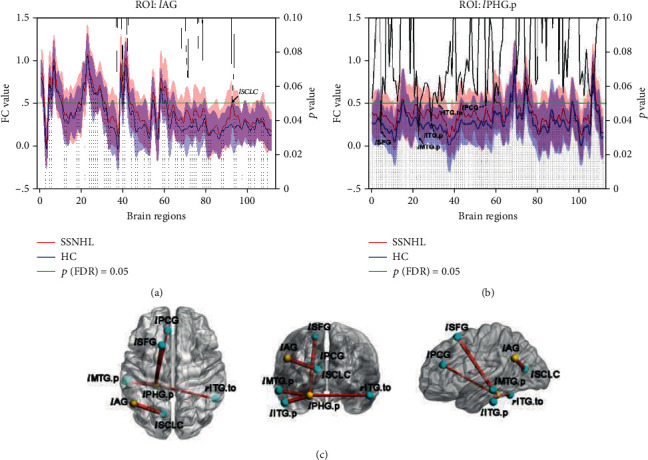
Intergroup comparison of ROI-wise FC between the SSNHL and HC groups. FC between the *l*AG and *l*SCLC was significantly increased (a). FCs between the *l*PHG.p and ROIs mainly located in the bilateral temporal lobes and left frontal lobe were significantly increased (b). The *x*-axis coordinates correspond to the numbers of the brain regions in the Harvard-Oxford Atlas. The *y*-axis coordinates on the left indicate the FC value, while those on the right indicate the *p* value. The red and blue curves indicate the mean FC values of the pair of ROIs in each group, and the shadow reflects the standard deviations. The black line indicates intergroup differences between each pair of ROIs, with a *p* value less than 0.05 where the black line is below the green horizontal line. ROI-wise FCs with significant difference between the SSNHL and HC groups are presented in a node and edge graph (c). The yellow balls indicate the seed ROI, while the cyan balls indicate the ROIs with significantly different FC values. The size of the sticks corresponds to the *t*-values from the two-sample *t*-tests. SCLC: supracalcarine cortex; PHG: parahippocampal gyrus; SFG: superior frontal gyrus; ITG: inferior temporal gyrus; PCG: paracingulate gyrus; *l*: left; p: posterior division; to: temporooccipital part.

Compared with the HC group, significantly increased FCs were observed in the SSNHL group between the left angular gyrus (*l*AG) and the left supracalcarine cortex (*l*SCLC) (Figures 3(a) and 3(c)), as well as between the posterior division of the left parahippocampal gyrus (*l*PHG.p) and ROIs mainly located in the bilateral temporal lobes and the left frontal lobe (Figures 3(b) and 3(c)).

To further investigate the brain functional alterations after hearing loss on different sides, the SSNHL patients were then divided into the left (L-SSNHL, *n* = 13) and right (R-SSNHL, *n* = 17) hearing loss subgroups for ROI-wise FC analyses. One-way ANOVA and post hoc analyses showed that the L-SSNHL subgroup displayed significantly increased FC between the *l*MTG.to and bilateral subcallosal cortex (SCC) compared to the HC group (Figure S1).

**Figure 4:**
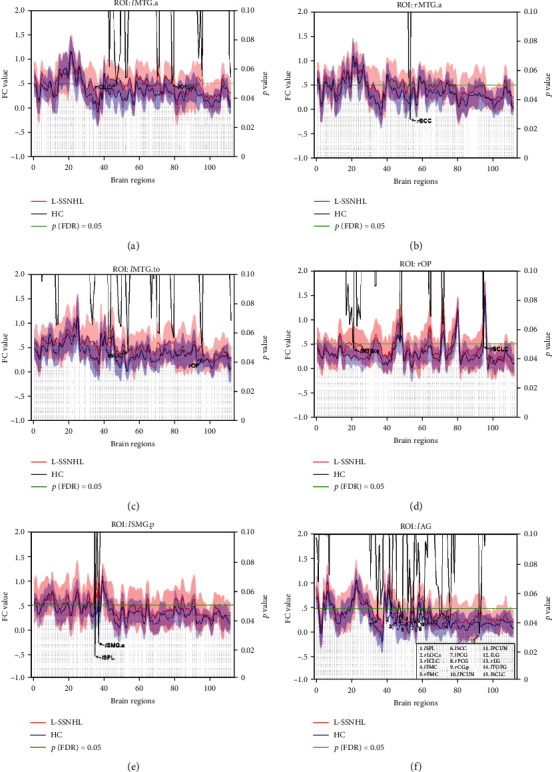
Intergroup comparisons of ROI-wise FC between the L-SSNHL and HC-1 subgroups. Compared with the HC-1 subgroup, the L-SSNHL subgroup showed significantly increased FCs between (a) the *l*MTG.a and the *r*ICLC and *l*OFG, (b) the *r*MTG.a and the *r*SCC, (c) the *l*MTG.to and the *r*SCC and *r*OP, (d) the *r*OP and the *l*MTG.a and *r*SCLC, (e) the *l*SMG.p and the *l*SPL and *l*SMG.a, and (f) the *l*AG and 15 ROIs indicated in the legend. MTG: middle temporal gyrus; OP: occipital pole; SMG: supramarginal gyrus; AG: angular gyrus; ICLC: intracalcarine cortex; OFG: occipital fusiform gyrus; SCC: subcallosal cortex; SCLC: supracalcarine cortex; SPL: superior parietal lobule; LOC: lateral occipital cortex; FMC: frontal medial cortex; PCG: paracingulate gyrus; CG: cingulate gyrus; PCUN: precuneus; LG: lingual gyrus; TOFC: temporal occipital fusiform cortex; *l*: left; *r*: right; a: anterior division; p: posterior division; s: superior division; to: temporooccipital part.

**Figure 5:**
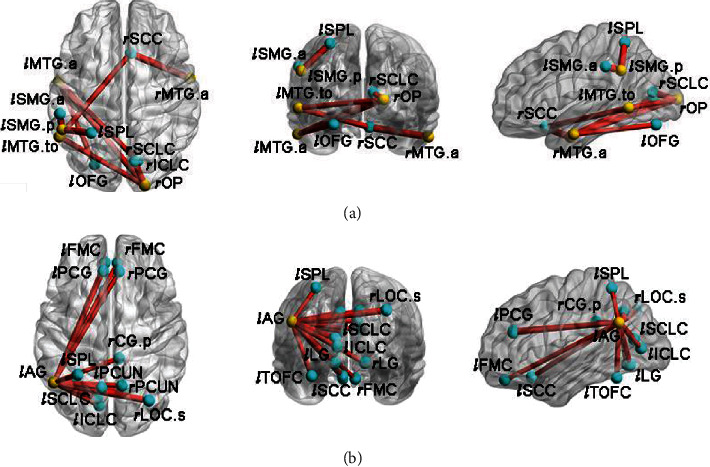
ROI-wise FC patterns with significant alterations between the L-SSNHL and HC-1 subgroups. Compared with the HC-1 subgroup, the L-SSNHL subgroup showed significantly increased FCs between the bilateral MTG and ROIs mainly located in the bilateral occipital lobes and the right limbic lobe, and between the *l*SMG.p and the left parietal cortex (a). Increased FCs were observed between the *l*AG and ROIs, mainly located in the bilateral frontal lobes, occipital lobes, and limbic lobes (b). The yellow balls indicate the seed ROI while the cyan balls indicate the ROIs with significantly different FC values. MTG: middle temporal gyrus; OP: occipital pole; SMG: supramarginal gyrus; AG: angular gyrus; ICLC: intracalcarine cortex; OFG: occipital fusiform gyrus; SCC: subcallosal cortex; SCLC: supracalcarine cortex; SPL: superior parietal lobule; LOC: lateral occipital cortex; FMC: frontal medial cortex; PCG: paracingulate gyrus; CG: cingulate gyrus; PCUN: precuneus; LG: lingual gyrus; TOFC: temporal occipital fusiform cortex; *l*: left; *r*: right; a: anterior division; p: posterior division; s: superior division; to: temporooccipital part.

Moreover, to better match the patients and healthy population, the HC group was also divided into two subgroups corresponding to the L-SSNHL and R-SSNHL subgroups, namely HC-1 (*n* = 13) and HC-2 (*n* = 17). Intergroup comparisons were performed between the L-SSNHL and HC-1 subgroups, as well as between the R-SSNHL and HC-2 subgroups using the two-sample *t*-test. Compared with the HC-1 subgroup, the L-SSNHL subgroup showed significantly increased FCs between the bilateral MTG and ROIs mainly located in the bilateral occipital lobes and the right limbic lobe (Figures 4(a)–4(d) and 5(a)). In addition, increased FCs were observed between the posterior division of the left supramarginal gyrus (*l*SMG.p) and ROIs located in the left parietal lobe (Figures 4(e) and 5(a)). Increased FCs were also observed between the *l*AG and ROIs mainly located in the bilateral frontal lobes, occipital lobes, and limbic lobes (Figures 4(f) and 5(b)).

We noticed that there were some inconsistencies in results derived from one-way ANOVA and two-sample *t*-test. We further compared the *p* values before and after FDR correction and found that these inconsistencies were mainly caused by the setting of the statistical threshold (Tables S1 and S2).

When using the NBS method for the correction of multiple comparisons, FCs were significantly increased between ROIs mainly located in the bilateral temporal lobes (*l*MTG.a, *l*MTG.p, *r*MTG.p, and *l*MTG.to) and occipital lobes (*l*LG, *r*LG, and *r*OP) in the L-SSNHL subgroup compared to the HC-1 subgroup (Figure S2). These results were also similar to those derived from FDR correction. However, SSNHL did not result in decreased FC in the SSNHL group or in the L-SSNHL subgroup. No significant differences were observed between the R-SSNHL and HC-2 subgroups.

## 4. Discussion

In this study, the patients with SSNHL showed changes in spontaneous neuronal activity and FC in multiple brain regions during the acute period of hearing loss. In the patients with SSNHL, ALFF and fALFF were increased in the bilateral putamen but decreased in the right MTG. FC was widely increased between brain regions including the auditory cortex, visual cortex, striatum, AG, PCUN, and limbic lobes.

Although the precise etiology of SSNHL has not been identified, several pathophysiological mechanisms have been postulated in previous studies. The potential causes included viral infections, vascular occlusion, endolymphatic hydrops, autoimmunity, neoplastic disease, ototoxic drugs, and head injury [3, 39]. The present study focused on spontaneous neuronal activity and FC in patients with SSNHL, which may contribute to improving the understanding of some clinical issues, such as speech comprehension impairments and the occurrence of emotional disorders in patients with SSNHL. A previous study reported a restoration of maximum speech discrimination scores (SDS) without an improvement in PTA in patients with SSNHL [40]. This phenomenon may be mediated by the remodeling of the cortex associated with speech comprehension and processing, which was consistent with the results of our FC analysis results. We hope that our discoveries will complement previous studies and help to explain the pathophysiological mechanisms of SSNHL.

In the SSNHL group, ALFF and fALFF values were increased in the bilateral putamen. This result suggested that SSNHL might lead to enhanced neuronal activity in the putamen, which is associated with the regulation of body movement and language processing [41–43]. A previous study showed that the activation of the putamen and caudate nucleus was increased in congenitally deaf adults when they were watching sign language [44]. As hearing loss causes difficulties in the perception and localization of sound, the brain may compensate for the function of language processing by increasing the activity of the putamen. Moreover, previous studies have shown that the STG had direct anatomical connections with the putamen and caudate nucleus [45]. This corticostriatal connection is related to spatial awareness and the encoding of the spatial locations of sounds [46, 47]. In the current study, patients with SSNHL showed increased FC values between the bilateral STG and left striatum, which may indicate alterations in the ability of sound localization and speech processing.

The MTG is associated with complicated sound processing, language comprehension, semantic memory, and integration of different types of information both within and across modalities (visual, auditory, or sensorimotor) [48–50]. In the present study, decreased ALFF and fALFF values in the right MTG suggested that the function of sound recognition and language processing was weakened due to insufficient acoustic stimulation in patients with SSNHL. In the SSNHL groups, the *r*MTG.p displayed increased FC with the bilateral LG and the right calcarine cortex, both of which are associated with visual function. Moreover, in the L-SSNHL subgroup, significantly increased FC values were also observed between the left MTG and visual cortical areas. These results indicated that cross-modal cortical reorganization occurred in patients with SSNHL, i.e., reduced acoustic stimulus input may have led to the transformation of cortical function from the auditory sense toward the visual sense in the MTG via cross-modal neuroplasticity. This finding was consistent with previous studies [51, 52]. In addition, our findings also indicated that cross-modal reorganization can occur within the acute period of SSNHL, which is consistent with a previous report [24].

The AG is located at the junction between the temporal, parietal, and occipital lobes. Given its rich anatomical connections to other brain structures, the AG is considered an essential hub for information integration of different modalities [53]. In the present study, the FC values in the L-SSNHL subgroup were increased between the *l*AG and 15 other brain regions, which are mainly located in visual cortical areas, limbic lobes, and the bilateral PCUN. Thus, hearing loss might lead to enhanced integration and processing of visual information, potentially representing a compensatory neural mechanism in which reduced auditory information inputs are compensated with an enhanced visual sense. In the limbic lobe, the SCC has been identified as an important brain area in emotional information processing [54]. The paracingulate gyrus (PCG) is involved in manipulating information concerning social interactions [55]. Previous studies reported an association between SSNHL and an increased incidence of affective disorders, including anxiety and depression [18, 19]. Based on the findings of our study and other researchers, we proposed that the increased FC between the *l*AG and limbic lobes is associated with cognitive alternations and emotional modulation after the occurrence of SSNHL. This finding suggested that some proportion of patients with SSNHL may require psychotherapy.

As one of the key nodes of the default mode netwok (DMN), the PCUN exhibited increased activation at rest [56]. The PCUN plays a critical role in the mental representation of sounds and the construction of multimodal sensory imagery [57]. In the present study, increased FC between the *l*AG and bilateral PCUN may have represented a compensatory enhancement of multimodal sensory perception in the absence of hearing.

This study has several limitations. First, the background noise produced by the fMRI scanner reached more than 100 dB SPL. Although earplugs were used during scanning, the noise inevitably stimulated the auditory system to some extent. Due to the hearing discrepancy between the patients and HC individuals, the activation of the brain by the scanner noise may differ. In future studies, we will attempt to more effectively reduce and shield the background noise. Second, since the sample size of the present study was relatively small, it was difficult to perform analyses of ALFF and voxel-wise FC in the L-SSNHL and R-SSNHL subgroups. Combining these two subgroups of patients may have obscured some findings. More studies with larger sample sizes are needed to further clarify the alterations in brain function in SSNHL. Third, mental assessment of the SSNHL patients was not performed in this study. Considering that some affective disorders have been associated with SSNHL, future studies are needed to provide more information about the underlying mechanisms.

## 5. Conclusion

Based on the results of our study, SSNHL caused alterations in the neuronal activity and FC of brain regions mainly including the striatum, auditory cortex, visual cortex, MTG, AG, precuneus, and limbic lobes within the acute period of hearing loss.

## Figures and Tables

**Table 1:** Demographic and clinical information of the SSNHL patients and HC individuals.

	SSNHL	HC	*p* value
Number (*n*)	30	30	—
Sex (male/female)	16/14	16/14	—
Age (year)	43.43 ± 14.62	42.57 ± 11.47	0.799
BMI (kg/m^2^)	24.15 ± 3.89	23.96 ± 2.70	0.827
Side of hearing loss (left/right)	13/17	—	—
Hearing loss duration (day)	11.60 ± 9.64	—	—
PTA of affected ear (dB HL)			
Before treatment	87.80 ± 29.32^∗^	—	—
After treatment	72.80 ± 36.01^∗^	—	<0.001
PTA of unaffected ear (dB HL)			
Before treatment	18.70 ± 11.51^∗^	—	—
After treatment	15.17 ± 6.28^∗^	—	0.043

All data are presented as the mean ± SD. Data with asterisks were compared with paired-sample Student's *t*-test methods. BMI: body mass index; PTA: pure-tone average.

**Table 2:** The peak MNI coordinates and intensity of brain clusters with significant intergroup differences in ALFF and fALFF.

	Brain region	MNI coordinates *x*, *y*, and *z*			Number of voxels	Peak intensity
Increased ALFF	L putamen	-30	6	0	707	4.953

Decreased ALFF	R calcarine cortex	12	-99	-3	194	-3.830
	R middle temporal gyrus	51	-57	-3	205	-4.452
	R precentral gyrus	30	-9	42	209	-4.392

Increased fALFF	R putamen	27	-12	18	372	4.528
	L putamen	-30	0	12	362	4.586

Decreased fALFF	R middle temporal gyrus	42	-57	-3	119	-4.131

L: left; R: right.

**Table 3:** The peak MNI coordinates and intensity of brain clusters with significant intergroup differences in voxel-wise FC values.

ROI	Brain region	MNI coordinates *x*, *y*, and *z*			Number of voxels	Peak intensity
*l*STG.p	L putamen/L caudate nucleus	-18	24	-3	397	5.174
*r*STG.p	L putamen/L caudate nucleus	-15	21	-3	328	4.147
*r*MTG.p	Bl lingual gyri/R calcarine	-18	-84	-6	700	3.486
*l*SMG.a	L inferior frontal gyrus	-21	30	-9	535	4.210
*l*AG	Bl calcarine cortices/L posterior cingulate cortex	-9	-66	9	1042	3.861
*l*LG	R cerebellum posterior lobe	30	-78	-45	592	4.021
	L caudate nucleus	-12	24	-3	647	5.312
	L middle frontal gyrus	-27	24	60	512	4.634
*r*LG	R cerebellum posterior lobe	30	-75	-42	590	4.068
	L middle frontal gyrus	-27	24	60	381	4.705
*l*PT	L caudate nucleus/L putamen	-15	21	-9	364	4.480
*l*OP	L caudate nucleus/L putamen	-6	3	-21	321	4.147

STG: superior temporal gyrus; MTG: middle temporal gyrus; SMG: supramarginal gyrus; AG: angular gyrus; LG: lingual gyrus; PT: planum temporale; OP: occipital pole; L or *l*: left; R or *r*: right; Bl: bilateral; a: anterior division; p: posterior division.

## Data Availability

The data used to support the findings of this study are available from the corresponding authors upon request.

## Supplementary Materials

Supplementary Materials Figure S1: intergroup comparison of ROI-wise FC between the L-SSNHL, R-SSNHL, and HC groups. One-way ANOVA and post hoc analyses showed that FCs between *l*MTG.to and *l*SCC and *r*SCC were significantly increased between the L-SSNHL and HC groups. In the upper panel, the *x*-axis coordinates correspond to the numbers of the brain region in the Harvard-Oxford Atlas. The *y*-axis coordinates on the left indicate the FC value, while the ones on the right indicate the *p* value. The red and blue curves indicate mean FC values of the pair of ROIs in each group, with the shadow refers to the standard deviations. The black line indicates intergroup differences of each pair of ROIs, where the black line below the green horizontal line indicates a*p* value less than 0.05. The lower panel shows ROI-wise FC patterns with significant differences. The yellow ball indicates the seed ROI, while the cyan balls indicates the ROIs with significantly different FC values. The size of the sticks corresponds to the *F* values of one-way ANOVA analysis. MTG: middle temporal gyrus; SCC: subcallosal cortex; *l*: left; *r*: right; to, temporooccipital part. Table S1: the one-way ANOVA results of intergroup comparison between the L-SSNHL, R-SSNHL, and HC groups. Table S2: the two-sample two-tailed*t*-test results of intergroup comparison between the L-SSNHL and HC-1 subgroups. Figure S2: intergroup comparison of ROI-wise FC between the L-SSNHL and HC-1 subgroups by network-based statistics (NBS) analysis. FCs were significantly increased between ROIs mainly located at the bilateral temporal lobes (*l*MTG.a, *l*MTG.p, *r*MTG.p, and *l*MTG.to) and occipital lobes (*l*LG, *r*LG, and *r*OP) between the L-SSNHL and HC-1 subgroups.
