# Supplementary material for: Altered Brain Activity and Functional Connectivity in Unilateral Sudden Sensorineural Hearing Loss
Source: Neural Plast. 2020 Sep 22;2020:9460364. doi: 10.1155/2020/9460364 (PMC7527900; doi:10.1155/2020/9460364)
Supplement: Supplementary Materials — Figure S1: intergroup comparison of ROI-wise FC between the L-SSNHL, R-SSNHL, and HC groups. One-way ANOVA and post hoc analyses showed that FCs between lMTG.to and lSCC and rSCC were significantly increased between the L-SSNHL and HC groups. In the upper panel, the x-axis coordinates correspond to the numbers of the brain region in the Harvard-Oxford Atlas. The y-axis coordinates on the left indicate the FC value, while the ones on the right indicate the p value. The red and blue curves indicate mean FC values of the pair of ROIs in each group, with the shadow refers to the standard deviations. The black line indicates intergroup differences of each pair of ROIs, where the black line below the green horizontal line indicates ap value less than 0.05. The lower panel shows ROI-wise FC patterns with significant differences. The yellow ball indicates the seed ROI, while the cyan balls indicates the ROIs with significantly different FC values. The size of the sticks corresponds to the F values of one-way ANOVA analysis. MTG: middle temporal gyrus; SCC: subcallosal cortex; l: left; r: right; to, temporooccipital part. Table S1: the one-way ANOVA results of intergroup comparison between the L-SSNHL, R-SSNHL, and HC groups. Table S2: the two-sample two-tailedt-test results of intergroup comparison between the L-SSNHL and HC-1 subgroups. Figure S2: intergroup comparison of ROI-wise FC between the L-SSNHL and HC-1 subgroups by network-based statistics (NBS) analysis. FCs were significantly increased between ROIs mainly located at the bilateral temporal lobes (lMTG.a, lMTG.p, rMTG.p, and lMTG.to) and occipital lobes (lLG, rLG, and rOP) between the L-SSNHL and HC-1 subgroups. [file 9460364.f1.docx]

## Supplementary Material


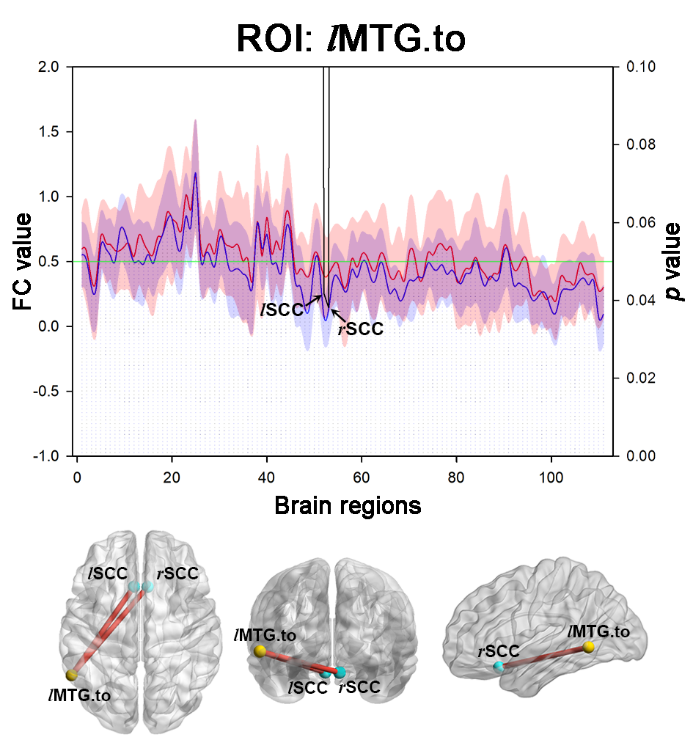


**Figure S1: Inter-group comparison of ROI-wise FC between** **L-SSNHL, R-SSNHL and HC groups.** One-way ANOVA and post hoc analyses showed that FCs between *l*MTG.to and *l*SCC, *r*SCC were significantly increased between L-SSNHL and HC groups. In the upper panel, the x-axis coordinates corresponded to the numbers of brain region in the Harvard-Oxford Atlas. The y-axis coordinates on the left indicated FC value while the ones on the right indicated *p* value. The red and blue curves indicated mean FC values of the pair of ROIs in each group, with the shadow referring to the standard deviations. The black line indicated inter-group differences of each pair of ROIs, with the *p* value less than 0.05 where the black line was below the green horizontal line. The lower panel showed ROI-wise FC patterns with significant differences. The yellow ball indicated the seed ROI while the cyan balls indicated the ROIs with significantly different FC values. The size of the sticks corresponded to the F values of One-way ANOVA analysis. MTG, middle temporal gyrus; SCC, subcallosal cortex; *l*, left; *r*, right; to, temporooccipital part.

**Table S1:** The one-way ANOVA results of inter-group comparison between L-SSNHL, R-SSNHL and HC groups.

| Seed ROI | Connected ROI | ANOVA | | t test | |
| --- | --- | --- | --- | --- | --- |
|  |  | *p* value | *p*FDR | *p* value | *p*FDR |
| *l*MTG.to | *l*SCC | < 0.001 | 0.041 | 0.002^#^ | 0.079 |
|  | *r*SCC | < 0.001 | 0.038 | 0.003^#^ | 0.047* |

The corresponding t test results of the significant FC found in ANOVA were showed as well. ^#^*p* < 0.05, **p*FDR < 0.05. MTG, middle temporal gyrus; SCC, subcallosal cortex; *l*, left; *r*, right; to, temporooccipital part.

**Table S2:** The two samples two-tailed t test results of inter-group comparison between L-SSNHL and HC-1 subgroups.

| Seed ROI | Connected ROI | t test | | ANOVA | |
| --- | --- | --- | --- | --- | --- |
|  |  | *p* value | *p*FDR | *p* value | *p*FDR |
| *l*MTG.a | *r*ICLC | 0.005 | 0.049 | 0.026^#^ | 0.319 |
|  | *l*OFG | 0.008 | 0.049 | 0.030^#^ | 0.322 |
| *r*MTG.a | *r*SCC | < 0.001 | 0.026 | 0.029^#^ | 1.000 |
| *l*MTG.to | *r*SCC | 0.003 | 0.047 | < 0.001^#^ | 0.038* |
|  | *r*OP | 0.004 | 0.043 | 0.044^#^ | 0.531 |
| *l*SMG.p | *l*SPL | < 0.001 | 0.015 | 0.008^#^ | 0.126 |
|  | *l*SMG.a | < 0.001 | 0.023 | 0.037^#^ | 0.456 |
| *l*AG | *l*SPL | 0.014 | 0.041 | 0.205 | 0.263 |
|  | *r*LOC.s | 0.001 | 0.048 | 0.012^#^ | 0.166 |
|  | *l*ICLC | 0.008 | 0.041 | 0.021^#^ | 0.140 |
|  | *l*FMC | 0.021 | 0.047 | 0.108 | 0.204 |
|  | *r*FMC | 0.017 | 0.041 | 0.083 | 0.184 |
|  | *l*SCC | 0.012 | 0.045 | 0.149 | 0.229 |
|  | *l*PCG | 0.016 | 0.041 | 0.175 | 0.249 |
|  | *r*PCG | 0.013 | 0.041 | 0.129 | 0.235 |
|  | *r*CG.p | 0.023 | 0.047 | 0.144 | 0.238 |
|  | *l*PCUN | 0.019 | 0.044 | 0.081 | 0.190 |
|  | *r*PCUN | 0.013 | 0.043 | 0.096 | 0.191 |
|  | *l*LG | 0.008 | 0.044 | 0.023^#^ | 0.105 |
|  | *r*LG | 0.006 | 0.047 | 0.032^#^ | 0.108 |
|  | *l*TOFC | 0.010 | 0.041 | 0.035^#^ | 0.101 |
|  | *l*SCLC | 0.001 | 0.032 | 0.003^#^ | 0.124 |
| *r*OP | *l*MTG.a | 0.001 | 0.046 | 0.009^#^ | 1.000 |
|  | *r*SCLC | 0.001 | 0.047 | 0.031^#^ | 1.000 |

The corresponding one-way ANOVA results of the significant FC found in t test were showed as well. ^#^*p* < 0.05, **p*FDR < 0.05. MTG, middle temporal gyrus; OP, occipital pole; SMG, supramarginal gyrus; AG, angular gyrus; ICLC, intracalcarine cortex; OFG, occipital fusiform gyrus; SCC, subcallosal cortex; SCLC, supracalcarine cortex; SPL, superior parietal lobule; LOC, lateral occipital cortex; FMC, frontal medial cortex; PCG, paracingulate gyrus; CG, cingulate gyrus; PCUN, precuneus; LG, lingual gyrus; TOFC, temporal occipital fusiform cortex; *l*, left; *r*, right; a, anterior division; p, posterior division; s, superior division; to, temporooccipital part.


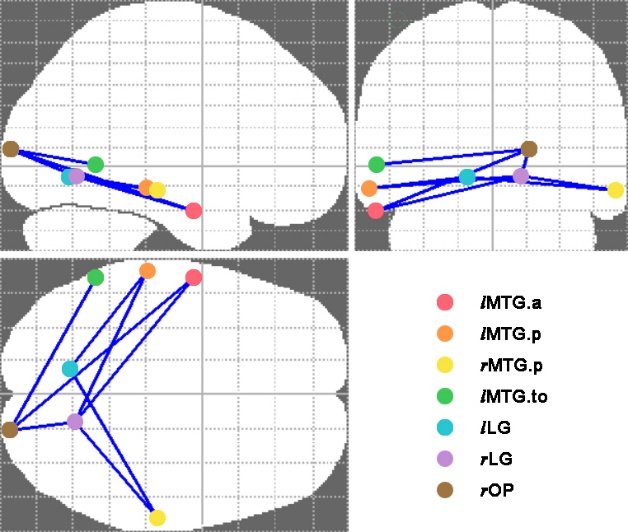


**Figure S2: Inter-group comparison of ROI-wise FC between L-SSNHL and HC-1 subgroups by network-based statistics (NBS) analysis.** FCs were significantly increased between ROIs mainly located at bilateral temporal lobes (*l*MTG.a, *l*MTG.p, *r*MTG.p and *l*MTG.to) and occipital lobes (*l*LG, *r*LG and *r*OP) between L-SSNHL and HC-1 subgroups.
